# Supplementary material for: Effect of the Application of Ultrasound to Homogenize Milk and the Subsequent Pasteurization by Pulsed Electric Field, High Hydrostatic Pressure, and Microwaves
Source: Foods. 2023 Mar 29;12(7):1457. doi: 10.3390/foods12071457 (PMC10093751; doi:10.3390/foods12071457)
Supplement: Supplementary file 1 [file foods-12-01457-s001.zip › foods-2289049-supplementary.pdf]

Supplementary material

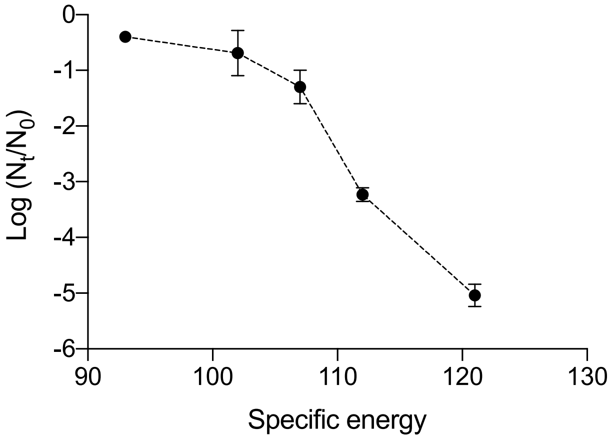

Figure S1. Inactivation of *Listeria monocytogenes* STCC 5672 in milk when applying a PEF treatment of 20 kV/cm and different specific energies (from 90 to 120 kJ/kg).
